# Aspirin Use Associated With Amyotrophic Lateral Sclerosis: a Total Population-Based Case-Control Study

**DOI:** 10.2188/jea.JE20140070

**Published:** 2015-02-05

**Authors:** Ching-Piao Tsai, Feng-Cheng Lin, Johnny Kuang-Wu Lee, Charles Tzu-Chi Lee

**Affiliations:** 1 Neurology, Neurological Institute, Taipei Veterans General Hospital and National Yang-Ming University, Taipei, Taiwan; 2 Department of Neurology, Kaohsiung Medical University Hospital, Kaohsiung Medical University, Kaohsiung, Taiwan; 3 Department of Neurology, Pingtung Hospital, Ministry of Health and Welfare, Taiwan; 4 General Education Center, University of Taipei, Taipei, Taiwan; 5 Department of Public Health, Kaohsiung Medical University, Kaohsiung, Taiwan

**Keywords:** amyotrophic lateral sclerosis, aspirin, case-control study

## Abstract

**Background:**

The association of aspirin use and nonsteroid anti-inflammatory drug (NSAID) use with amyotrophic lateral sclerosis (ALS) risk is unclear. This study determined whether use of any individual compound is associated with ALS risk by conducting a total population-based case-control study in Taiwan.

**Methods:**

A total of 729 patients with newly diagnosed ALS who had a severely disabling disease certificate between January 1, 2002, and December 1, 2008, comprised the case group. These cases were compared with 7290 sex-, age-, residence-, and insurance premium-matched controls. Drug use by each Anatomical Therapeutic Chemical code was analyzed using conditional logistic regression models. False discovery rate (FDR)-adjusted *P* values were reported in order to avoid inflating false positives.

**Results:**

Of the 1336 compounds, only the 266 with use cases exceeding 30 in our database were included in the screening analysis. Without controlling for steroid use, the analysis failed to reveal any compound that was inversely associated with ALS risk according to FDR criteria. After controlling for steroid use, we found use of the following compounds to be associated with ALS risk: aspirin, diphenhydramine (one of the antihistamines), and mefenamic acid (one of the NSAIDs). A multivariate analysis revealed that aspirin was independently inversely associated with ALS risk after controlling for diphenhydramine, mefenamic acid, and steroid use. The inverse association between aspirin and ALS was present predominately in patients older than 55 years.

**Conclusions:**

The results of this study suggested that aspirin use might reduce the risk of ALS, and the benefit might be more prominent for older people.

## INTRODUCTION

Amyotrophic lateral sclerosis (ALS) is a fatal neurodegenerative disease. Without mechanical ventilation, death resulting from respiratory failure typically follows within 2 to 5 years of the onset of symptoms.^1^ An increase in the prevalence and incidence of ALS has been observed in Hong Kong,^2^ Japan,^3^ Sweden,^4^ and possibly worldwide.^5^^,^^6^ The only medication used for treatment, riluzole, improves the quality of life and survival of patients with ALS^7^^–^^9^ but is not a cure.

Substantial evidence has implicated both inflammation and mitochondrial dysfunction in ALS pathogenesis.^10^^–^^13^ Inhibitors of the pro-inflammatory enzyme cyclooxygenase (COX)-2, traditional nonaspirin, nonsteroid, anti-inflammatory drugs (NSAID), significantly delay the onset of motor dysfunction in the SOD1-G93A transgenic mouse model of ALS.^14^ Besides, a meta-analysis that included 5 large prospective cohort studies revealed that neither NSAID nor aspirin use was associated with overall ALS risk.^15^ Treatment of ALS with anti-inflammatory drugs (to prevent the disease or slow its progression) has yielded mixed results, despite evidence indicating that local cytotoxic inflammation occurs in ALS.^16^ Groups of drugs that are similar to NSAIDs exert heterogeneous effects, and an individual compound might contribute to ALS.^15^ Therefore, we conducted a total population-based case-control study to screen individual compounds that might potentially contribute to ALS risk in Taiwan.

## MATERIAL AND METHODS

### National Health Insurance in Taiwan

In 1995, the National Health Insurance (NHI) program, a government-run single-payer insurance system, was established in Taiwan. By December 2010, 23.074 million people were enrolled nationwide, with a coverage rate of 99.6%. The registration of all cases of severely disabling diseases (SDDs), such as chronic renal failure, myasthenia gravis, cancer, and ALS, is required by the NHI Bureau before SDD certification can be granted. In 2008, 37 099 medical doctors and 553 neurology specialists were registered in Taiwan. In addition, 790 621 people had SDD certificates in 2008, constituting 3.4% of the total population.

### Sample

This was a total population-based case-control study. The National Health Insurance Research Database (NHIRD), which contains outpatient, ambulatory, hospital inpatient care, and dental records, was used in this study. Prescriptions for individual compounds were double-checked by a physician and a pharmacist, and the NHI covered almost all residences; therefore, any information bias related to prescribed compounds should be minimal. The ALS cases were identified by International Classification of Diseases, Ninth Revision (ICD-9) code number 335.20. The study period that included incident ALS patients (2002–2008) was dependent on all SDD and NHIRD data published since 1996. The medical claims data from 1996 to 2001 were used to verify that the ALS patients in this study were new cases. The ALS diagnosis was ascertained based on El Escorial criteria by an in-charge clinical neurologist, and the medical records of patients were sent to the NHI Bureau. Another group of neurologists of the NHI Bureau used El Escorial criteria to verify the medical records of ALS to confirm the diagnosis.^17^ All ALS patients met El Escorial criteria in this study. Only ALS patients with SDD certification were included. Patients with SDD certificates were eligible for exemption from insurance premiums and copayments. The approval of SDD certificates requires a strict evaluation by the Executive Yuan’s Department of Health in Taiwan. In this study, all ALS cases were verified by linking encrypted identification numbers with SDD certificates.

Overall, 729 new ALS cases from January 1, 2002, to December 31, 2008, were included in this study. These cases were compared with 7290 sex-, age-, residence-, and insurance premium-exact-matched controls. Because of the exact-matched design, sex, residence, and the insurance premium category were equivalent between cases and their controls. Controls were further collected with birthdays as close as possible to their corresponding cases. Residence was categorized as rural or urban. The insurance premium was a proxy indicator of economic status and was classified into 1 of 4 categories: fixed premium and dependent, New Taiwan Dollar (NTD) <20 000 monthly, NTD 20 000–39 999 monthly, and NTD ≥40 000 monthly (1 United States Dollar = 32.1 NTD in 2008). The fixed premium group was composed of people receiving social welfare support and included veterans and low-income earners. The dependent-insurance premium group was composed of people with family members who did not have a job or income. The analysis data were provided by the NHIRD without personally identifiable information, so approval by the institutional review board was unnecessary.

### Statistical analyses

Chi-square tests were used to examine the differences in the demographic characteristics and steroid use between the newly diagnosed ALS cases and control patients. Drug use by each Anatomical Therapeutic Chemical code^18^ (ATC-code) during the 2 to 5 years prior to ALS diagnosis date was analyzed using a conditional logistic regression model adjusted for steroid use. To evaluate whether individual drugs were an independent risk factor for developing ALS, we excluded drug use in the year prior to diagnosis. The defined daily dose (DDD) recommended by the World Health Organization (WHO) is a unit for assessing drug dose on a standard scale. Cumulative DDD (cDDD), which indicates the exposed duration, was estimated as the sum of dispensed DDD of the drug to compare its use with the risk of ALS. Steroid use was quantified using the cDDD for 1 to 5 years prior to ALS diagnosis and served as a proxy indicator of inflammation severity. Steroid use was classified into 1 of 3 categories: 0, 1–29, and ≥30. The cut-off point of 30 was the third quartile of steroid use in our study patients. The false discovery rate^19^ (FDR)-adjusted *P* value, which has been widely used in multiple testing studies, was reported in order to avoid inflating false positives.

Of 1336 potential compounds, only 266 were included in the screening analysis because their use cases exceeded 30 in our database. We first tested each of these compounds individually without controlling for steroid use. We subsequently analyzed the use of these drugs but controlled for steroid use as a proxy indicator of inflammation severity. In the third step, use of the candidate compounds was analyzed using multivariate conditional logistic regression to reveal the potential independent factors. The model was first tested among the total group of patients, then among subgroups according to sex and age (15–54 years and ≥55 years). Analyses were conducted using SAS Version 9.3 (SAS Institute Inc., Cary, NC, USA).

### Sensitivity analyses

To validate the robustness of the primary study findings, we performed several sensitivity analyses. First, we defined the usage of potential compounds as cDDD, classified into 1 of 3 categories: 0 cDDD, 1 cDDD through third quartile, and ≥ third quartile. The cut-off point was the third quartile of each drug use in our study patients. In addition, to investigate the interaction between the main study compound and steroid use on the risk of ALS, subgroups were analyzed separately by nonsteroid- and steroid-using patients. Because subgroup data of nonsteroid- and steroid-using patients did not maintain matching between ALS and non-ALS patients, we analyzed these subgroups using unconditional logistic regression. Finally, we added past history of diseases into the regression model to further control for sampling bias. The potential confounding diseases were cerebrovascular disease (ICD-9:433–439), ischemic heart disease (410–414), and peripheral vascular disease (443.9).

## RESULTS

### Sample characteristics

The demographic and clinical characteristics of patients are summarized in Table 1. Because of the matched design, the age, sex, residence, and insurance-premium distributions of the ALS and non-ALS groups were equivalent. The mean (± standard deviation [SD]) age of the ALS patients was 57.43 ± 13.09 years, and that of control patients was 57.43 ± 13.09 years (*P* = 0.999, *t* test). Among the 729 new ALS patients, with 451 men and 278 women, and the male-to-female ratio was 1.6. One fifth of patients lived in rural areas. Nearly half of the cases were listed as receiving social support or as a dependent member of a family. Approximately 15% of ALS patients reported aspirin use for 2 to 5 years prior to ALS diagnosis, while in the same period, approximately 19% of the non-ALS patients reported aspirin use. The rate of aspirin use in ALS patients was significantly lower than in non-ALS patients (*P* = 0.019). Approximately 57% of ALS patients and 50% of non-ALS patients reported steroid use. The mean cumulative dose for all aspirin-use patients was 2.45 ± 4.51 cDDD. The mean cumulative dose for all steroid-use patients was 42.33 ± 136.06 cDDD.

**Table 1.** Demographic and clinical characteristic of amyotrophic lateral sclerosis (ALS) and matched non-ALS patients in Taiwan, 2002–2008

Characteristic	ALS, *n* = 729 (%)	Non-ALS, *n* = 7290 (%)	Chi-square *P* value
Age at baseline (years)			
15–54	320 (43.90)	3200 (43.90)	1.000
≥55	409 (56.10)	4090 (56.10)	
Gender			
Female	278 (38.13)	2780 (38.13)	1.000
Male	451 (61.87)	4510 (61.87)	
Residence			
Rural	154 (21.12)	1540 (21.12)	1.000
Urban	575 (78.88)	5750 (78.88)	
Insurance premium			
Fixed premium and dependent	363 (49.79)	3630 (49.79)	1.000
NTD^a^ <20 000	189 (25.93)	1890 (25.93)	
NTD 20 000–39 999	132 (18.11)	1320 (18.11)	
NTD ≥40 000	45 (6.17)	450 (6.17)	
Steroid use			
No	310 (42.52)	3660 (50.21)	<0.001
Yes	419 (57.48)	3630 (49.79)	

^a^1 United States Dollar = 32.1 New Taiwan Dollars (NTD) in 2008.

### Compounds inversely associated with ALS incidence

Without controlling for steroid use, the analysis failed to demonstrate that any compound was inversely associated with ALS risk according to FDR-adjusted criteria. After controlling for steroid use, the following compounds potentially contributed to risk of ALS: aspirin, diphenhydramine (an antihistamine), and mefenamic acid (an NSAID) according to FDR-adjusted criteria (Table 2). A multivariate analysis revealed that aspirin was independently inversely associated with ALS risk after controlling for diphenhydramine, mefenamic acid, and steroid use (Table 3). Compared with the no-aspirin-use group, the adjusted odds ratio was 0.69 (95% confidence interval [CI], 0.56–0.87) for the group with any aspirin use. The adjusted conditional logistic regression results revealed that diphenhydramine and mefenamic acid use had a nonsignificant association with ALS incidence when controlling for aspirin and steroid use.

**Table 2.** Top 20 compounds having an inverse association with amyotrophic lateral sclerosis (ALS)

ATC-code	Compound name	*P* value		Odds-ratio (95% CI)	Number of drug use patients (%)	
	
		Raw	FDR		ALS	Non-ALS
N02BA01	Acetylsalicylate (Aspirin)	0.0004	0.0173*	0.67 (0.54, 0.84)	111 (15.23%)	1369 (18.78%)
R06AA02	Diphenhydramine	0.0013	0.0323*	0.68 (0.54, 0.86)	94 (12.89%)	1150 (15.78%)
M01AG01	Mefenamic Acid	0.0016	0.0358*	0.77 (0.66, 0.91)	384 (52.67%)	4134 (56.71%)
R05DB24	Tipepidine Hibenzate	0.0027	0.0517	0.66 (0.50, 0.87)	64 (8.78%)	857 (11.76%)
A02BA01	Cimetidine	0.0029	0.0521	0.77 (0.65, 0.92)	234 (32.1%)	2564 (35.17%)
R03CC03	Terbutaline Sulfate	0.0038	0.0630	0.62 (0.45, 0.86)	42 (5.76%)	599 (8.22%)
R03DA05	Aminophylline	0.0044	0.0682	0.73 (0.59, 0.91)	124 (17.01%)	1383 (18.97%)
C09AA02	Enalapril Maleate	0.0052	0.0726	0.64 (0.46, 0.87)	46 (6.31%)	643 (8.82%)
R06AX15	Mebhydroline	0.0059	0.0748	0.73 (0.58, 0.91)	97 (13.31%)	1155 (15.84%)
R06AB04	Chlorpheniramine Mal	0.0058	0.0748	0.75 (0.61, 0.92)	128 (17.56%)	1455 (19.96%)
C09AA01	Captopril	0.0070	0.0810	0.63 (0.45, 0.88)	41 (5.62%)	560 (7.68%)
R06AA07	Piprinhydrinate	0.0080	0.0862	0.70 (0.54, 0.91)	70 (9.6%)	868 (11.91%)
A02AF02	Simethicone Emulsion	0.0086	0.0862	0.77 (0.63, 0.94)	562 (77.09%)	5762 (79.04%)
N02BE01	Acetaminophen	0.0108	0.0992	0.76 (0.61, 0.94)	605 (82.99%)	6183 (84.81%)
R06AX12	Terfenadine	0.0120	0.1068	0.78 (0.65, 0.95)	153 (20.99%)	1716 (23.54%)
J01CA04	Amoxycillin	0.0127	0.1091	0.82 (0.70, 0.96)	392 (53.77%)	4099 (56.23%)
R05DB01	Benzonatate	0.0136	0.1097	0.67 (0.49, 0.92)	46 (6.31%)	591 (8.11%)
G04BX06	Phenazopyridine	0.0145	0.1104	0.65 (0.46, 0.92)	38 (5.21%)	524 (7.19%)
R05CA10	Glycyrrhiza Fluid Ext	0.0173	0.1214	0.74 (0.58, 0.95)	83 (11.39%)	973 (13.35%)
C01DA08	Isosorbide Dinitrate	0.0190	0.1294	0.63 (0.43, 0.93)	30 (4.12%)	413 (5.67%)

ATC-code, Anatomical Therapeutic Chemical code; CI, confidence interval; FDR, false discovery rate.

*FDR-adjusted *P* < 0.05.

**Table 3.** Association between aspirin, diphenhydramine, and mefenamic acid use and amyotrophic lateral sclerosis (ALS) risk

Variable	Unadjusted analysis		Adjusted analysis	
	
	Odds ratio (95% CI)	*P* value	Odds ratio (95% CI)	*P* value
Aspirin^a^	0.67 (0.54–0.84)	<0.001	0.69 (0.56–0.87)	0.001
Diphenhydramine^a^	0.68 (0.54–0.86)	0.001	0.87 (0.72–1.04)	0.132
Mefenamic Acid^a^	0.77 (0.66–0.91)	0.002	0.96 (0.80–1.16)	0.695
Steroid (cDDD)				
0	1.00		1.00	
1–29	1.17 (0.98–1.39)	0.078	1.25 (1.04–1.49)	0.015
≥30	2.11 (1.70–2.62)	<0.001	2.38 (1.90–2.99)	<0.001

cDDD, cumulative defined daily dose; CI, confidence interval.

^a^Any use vs. no use.

### Stratified analyses

Table 4 lists the analysis among subgroups according to sex and age (15–54 years and ≥55 years). After controlling for steroid, diphenhydramine, and mefenamic acid use, aspirin exhibited an independent inverse association with ALS risk among women older than 55 years of age (OR 0.60; 95% CI, 0.37–0.98) and among men older than 55 years of age (OR 0.70; 95% CI, 0.49–0.99).

**Table 4.** Stratified analyses of the association between aspirin use and amyotrophic lateral sclerosis (ALS) risk

Gender by age group	Adjusted odds ratio (95% CI)	*P* value
Male		
15–54 years	0.88 (0.57–1.37)	0.583
≥55 years	0.70 (0.49–0.99)	0.047
Female		
15–54 years	0.72 (0.47–1.11)	0.077
≥55 years	0.60 (0.37–0.98)	0.040

CI, confidence interval.

### Sensitivity analyses

After categorizing usage of potential compounds as cDDD, aspirin use was inversely associated with ALS. Compared with the group of people who did not use aspirin, the adjusted odds ratios were 0.68 (95% CI, 0.51–0.90) for the group prescribed aspirin below 2.5 cDDD (the third quartile in this study) and 0.73 (95% CI, 0.54–1.00) for the group with a cumulative aspirin use exceeding 2.5 cDDD. Moreover, the analysis subgroup of patients not using steroids showed that aspirin was not associated with ALS risk (OR 0.91; 95% CI, 0.58–1.44) when controlling for diphenhydramine and mefenamic acid use. In contrast, among patients who used steroids, aspirin was associated with ALS risk (OR 0.77; 95% CI, 0.59–0.99) when controlling for diphenhydramine and mefenamic acid use. After controlling for cerebrovascular disease, ischemic heart disease, and peripheral vascular disease, the inverse association between aspirin and ALS (OR 0.68; 95% CI, 0.53–0.84) was similar to that observed in the main analysis.

## DISCUSSION

This study is the first to screen the association between individual prescription compounds and ALS risk in a population-based study. Using data from the total population of Taiwan, our findings indicated that the incidence of ALS was inversely associated with aspirin use when controlling for diphenhydramine, mefenamic acid, and steroid use.

Previous studies have employed both case-control and cohort approaches to suggest the effects of aspirin on the reduced risks of prostate cancer^20^ and colon cancer.^21^ Randomized trials of daily aspirin use versus no aspirin have also been suggested to assess the effectiveness of aspirin in preventing cancer incidence and mortality.^22^^–^^24^ For the study of aspirin and ALS, aspirin treatment delays the appearance of reflex, coordination, and muscle strength deficits in the mouse model of ALS if the treatment begins sufficiently early.^25^ Although free radicals and inflammation constitute major routes of neuronal injury occurring in ALS, Shin et al (2012) developed 2-hydroxy-5-[2-(4-trifluoromethylphenyl)-ethylaminobenzoic acid] (AAD-2004) as a derivative of aspirin and showed that, compared to riluzole, this compound reduced autophagosome formation, axonopathy, and motor-neuron degeneration while improving motor function and increasing life span.^26^

Although ALS risk was not associated with aspirin use in several observational studies,^15^^,^^27^ aspirin was inversely associated with ALS incidence in this study. The observed differences between the inverse association found in this study and previous studies that observed no association could be caused by differences in controlling for confounders. Without controlling for steroid use, we observed a nonsignificant association between aspirin and ALS. When considering steroid-dose usage, aspirin use and ALS showed an inverse association. Moreover, a significant inverse association exists between aspirin use and ALS among steroid users but not among nonsteroid users. Because steroid use was considered the proxy variable of inflammation severity, controlling for a certain type of inflammation severity index might help pinpoint which drugs contribute to ALS in an observational study. The interaction between aspirin and prednisolone (a steroid) use was mildly antagonistic in a study of the treatment of rheumatoid arthritis.^28^ Although using aspirin together with a steroid may make aspirin less effective, we still found a significant inverse association between aspirin use and ALS among steroid users after controlling for demographic variables and diphenhydramine and mefenamic acid use. The effectiveness of co-administering aspirin and steroids for the potential prevention of ALS is still unclear and needs further study.

Mean age of the ALS and control patients in this study was around 57 years; therefore, we performed subgroup analysis according to age (15–54 years and ≥55 years) using a cut-off point of 55 years for interpretation purposes. Aspirin use exhibited an independent inverse association with ALS risk among men and women aged ≥55 years, but we did not observe a significant association among patients aged 15–54 years. One possible explanation of this discrepancy may be the longer duration of aspirin use in older people compared to the younger age group. Benefits of aspirin chemoprevention of colorectal cancer were more evident when aspirin was used at a high dose and for periods longer than 10 years.^29^ However, a randomized controlled trial failed to demonstrate a reduction in colorectal cancer incidence with aspirin use at 10-year follow-up.^30^ For potential prevention of ALS, the age at which aspirin use begins and the appropriate duration and dose are still unclear and need further study.

The results of previous epidemiological studies have not been uniform, and questions remain regarding the mechanism through which aspirin may affect ALS occurrence. Thus, focused clinical trials and further epidemiological studies and laboratory investigations are necessary to define the potential of aspirin to prevent ALS.

On conducted a screen analysis, use of all top 20 compounds exhibited an inverse association with ALS when assessed without controlling for the effect of multiple comparisons. However, after considering the multiple-comparison problem and adjusting using the FDR method, only aspirin, diphenhydramine, and mefenamic acid use were significantly associated with ALS. Because FDR is a multiple-comparison adjustment method, this method provided the candidate compounds best suited for further consideration. Therefore, similar to the current investigation of aspirin, the potential contributions of other compounds are worthy of further study.^31^^,^^32^

The major strength of this study is its large size. As far as we know, this is the first national population study to explore the association between aspirin use and ALS and the first to fully screen individual compounds for the association between use of these compounds and risk of ALS. This study was implemented using the total population of Taiwanese citizens seen in medical practice; therefore, the conclusions are likely to be applicable to the general population.

However, this study also had several limitations. In epidemiological studies, the time of the first symptom onset is epidemiologically the most relevant. However, our study database did not contain information on first symptom onset. The database also did not contain certain useful predictors, such as ALS symptoms, or any information on cigarette smoking, which is a crucial confounding factor. In addition, other confounding factors, such as past history of illness, complications, and symptoms may also lead to bias in this study. Another limitation of this study is that ALS is a rare disease in Taiwan, and although this was a 7-year total population-based study, only 729 new ALS cases were verified. This was an observational population-based study; therefore, we recommend a randomized clinical trial in a country with a higher incidence of ALS in order to recruit a sufficient number of patients to confirm our findings. In summary, our study suggests the potential role of aspirin in preventing ALS.
